# “CLUPS”: A New Culture Medium for the Axenic Growth of* Entamoeba histolytica*

**DOI:** 10.1155/2018/2796516

**Published:** 2018-07-11

**Authors:** F. Gonzalez-Salazar, I. Meester, F. J. Guzmán De La Garza, L. H. De La Garza-Salinas, A. Sampayo-Reyes, J. N. Garza-Gonzalez, O. Monsivais-Diaz, B. A. Barba-Dávila, M. E. Hernández-García, J. Vargas-Villarreal

**Affiliations:** ^1^Centro de Investigación Biomédica del Noreste, Instituto Mexicano del Seguro Social, Monterrey, Mexico; ^2^Departamento de Ciencias Básicas, Vicerrectoría de Ciencias de la Salud, Universidad de Monterrey, Monterrey, Mexico; ^3^Coordinación de Planeación y Enlace Institucional, Instituto Mexicano Del Seguro Social, Delegación Nuevo León, Monterrey, Mexico; ^4^Departamento de Inmunología y Virología, Universidad Autónoma de Nuevo León, San Nicolás de los Garza, Mexico; ^5^Escuela de Ingeniería y Ciencias, Instituto Tecnológico de Estudios Superiores de Monterrey, Monterrey, Mexico

## Abstract

Amebiasis remains a major health problem in Mexico. Therefore, the search for better culture media and low-cost diagnostic and therapeutic tools is fundamental. We present a new culture medium for* Entamoeba histolytica* which allows the microbe to preserve its virulence factors and ability to induce hepatic abscesses in animal models. The novel CLUPS medium is an improved version of the PEHPS medium, previously designed in our laboratory. The main difference is the substitution of raw beef liver in PEHPS by raw beef lung in the CLUPS medium. To compare the performance of three-culture media (traditional TYI-S-33, PEHPS, and CLUPS),* E. histolytica* trophozoites were cultured in quintuplicate, followed by the evaluation of phospholipase activity and the induction of liver abscesses in golden hamsters.* E. histolytica* trophozoites grew significantly better in CLUPS medium than in TYI-S-33. Likewise, CLUPS-cultured trophozoites produced significantly more phospholipases than TYI-S-33-cultured trophozoites. Finally, trophozoites grown in any of the three tested media had similar potential to induce liver abscesses.

## 1. Introduction

Amebiasis is one of the main causes of infectious disease in Mexico and African countries. The World Health Organization reported in 1997 that there were about 40 million disease cases worldwide per year and estimated that over 400 million people were asymptomatically infected [1, 2]. Unfortunately, there are no more recent world reports.

On the other hand, since the start of this century, novel nonpathogenic* Entamoeba* species have been recognized [3, 4]. Ongoing improvement of molecular diagnostic tools facilitates a better differentiation and identification of species [5]. The incorporation of new diagnostic techniques confirms that the protozoan pathogen* Entamoeba histolytica* remains a major cause of gastrointestinal disease in Mexico and other parts of the world [6, 7]. PCR-based studies reveal that* E. histolytica* is present in 16-20% of people with a gastrointestinal disorder [6, 7]. Better culture media could facilitate the* in vitro* study of microorganisms such as* E. histolytica*. Ideally, cultured parasites should present the same growth and virulence characteristics as their counterparts in their natural habitat [8]. Axenic cultivation of* E. histolytica* was first accomplished by Diamond in 1961 [9], using a diphasic medium composed of a serum-enriched agar base covered by a broth supplemented with chicken extract and vitamins. In 1968, Diamond described a new monophasic medium called TPS-1 [10] that received great acceptance among researchers who cultivated amebas. In 1978, a new medium was introduced that contained a yeast extract instead of Panmede (a papain digest of ox liver) [11]. This new medium, TYI-S-33, became the most popular [11]. A casein-free alternative, YI-S, has never been widely used [12, 13]. In 1988, our group reported an in-house developed axenic culture medium, PEHPS [14]. PHEPS is based on TPS-1 but replaces Panmede with the pancreatic digest of raw beef liver as described in [14]. The main components of the* E. histolytica* axenic culture media are a source of peptides, amino acids (trypticase or casein digest peptone), nucleic acids (yeast extract), carbohydrates (glucose), lipids (serum), and vitamins. The development of a new culture medium for intestinal protozoan allows learning about the biology of parasites, their growth rate, virulence factors (like phospholipase activity), susceptibility to new drugs, and the appearance of resistant strains, among other aspects [15].

The PEHPS medium was designed because of the need of an economic medium without the interlot variability present in manufactured culture media. In the commercial media TYI-S-33 and TPS, the supplements, Panmede, and yeast extract present batch-to-batch variability [16], whereas our in-house PEHPS is produced as a single lot that can take up to a year to be consumed [14]. The main components of PEHPS are casein peptone, liver and pancreas extract, and bovine serum [14]. Bovine serum is essential and cannot be substituted; trophozoites died at day three in serum-free cultures [17]. However, the inconvenience of beef extracts nowadays is that remnants of antibiotics and antiparasitic drugs may be present, as has been proven for the liver of cattle [18, 19]. We previously demonstrated that ivermectin and similar antiparasitic drugs inhibit the* in vitro* growth of* E. histolytica* [20].

The main objective of this study was to demonstrate that the new CLUPS medium facilitates proper* E. histolytica* growth without affecting virulence factors nor the ability to cause hepatic abscesses in animal models.

## 2. Material and Methods

### 2.1. Biologic Material

Beef liver, lungs, and pancreas and pork pancreas were obtained at a local slaughterhouse from recently sacrificed animals. All organs were transported in ice and manipulated as previously described [14]. Extracts were frozen at -20°C until use.

### 2.2. Medium Preparation

The composition and preparation of CLUPS medium are the same as those of PEHPS medium [14], except for the substitution of beef liver (PEHPS) by beef lung (CLUPS). The main components of CLUPS are casein, lung and pancreas extract, and bovine serum.  Table 1 compares the composition of the three media, PEHPS, TYI-S-33, and CLUPS. Once prepared, the basal medium was sterilized by filtration over a 0.22-*μ*m filter (Millipore, Millipore Corporation, Billerica, MA, USA) and stored in 5.5-mL aliquots at -20°C. Just before use, CLUPS is supplemented with 8.3% v/v bovine serum. Bovine serum was also produced in-house as follows: about 15 L fresh blood from cows for human consumption was collected in a sterile way at a local slaughterhouse. After blood coagulation, the serum was recovered, and the complement was heat-inactivated (56°C, 1 h). Next, the serum was prefiltered through Whatman No. 1 paper before being sterile-filtered through a series of sterile Millipore HAWP filters (Millipore, Millipore Corporation, Billerica, MA, USA) from 5 to 0.22 *μ*m. Serum was frozen at -70°C until use.

### 2.3. Culture Strains

Trophozoites from the* E. histolytica* HM1: IMSS strain were cultured in TYI-S-33 medium under axenic conditions. The reference strain was maintained in 13 × 100 mm borosilicate screw-capped tubes containing 5.5 mL TYI-S-33 medium plus 0.5 mL bovine serum. Each tube was inoculated with 1 × 10^4^ trophozoites/mL and incubated at 36.5°C for 72 h until the amebas were harvested as described [21]. Ameba density was determined with a hemocytometer according to López-Revilla and Rodríguez-Báez [22] before being reseeded.

For assays, the parasites were maintained in axenic conditions at 36.5°C for 30 days by serial subcultivation in TYI-S-33, PEHPS, or CLUPS medium.

Growth curves of* E. histolytica* in TYI-S-33, PEHPS, or CLUPS medium were determined in quintuplicate every 24 h for 5 days. A hemocytometer was used to determine the number of trophozoites/mL, and the doubling time was calculated by linear regression [23]. The yields in each culture were determined at the end of the logarithmic growth phase (72 h of incubation).

### 2.4. Collection Phospholipase-Containing Subcellular Fraction

Phospholipase A activity can be recovered from fraction P30 using a previously described cellular fractionation method [24]. Cultured protozoa was harvested and washed twice with phosphate-buffered saline, pH 7.0 by centrifugation (1500* x g*, 10 min). The protozoa was resuspended and diluted in 2 volumes of Hank's balanced salt solution.Next, the trophozoites were homogenized using a motor-driven Elvehjem-Potter Teflon/glass homogenizer (100 strokes) to obtain the “total extract”. The “total extract” was centrifuged at 30,000* x g* for 10 min. Both the supernatant (S30) and the precipitate (P30) were stored in 200-*μ*l aliquots at -70°C until use.

### 2.5. Detection of Phospholipase A Activity

The phospholipase activity of types A_1_ and A_2_ was determined according to Vargas-Villarreal et al. [24]. The assay chromatographically analyzes radioactive hydrolysis products, either free fatty acids (FFA) or lysophosphatidylcholine (LPC), of 2-palmitoyl-(2-palmitoyl-^14^C)-phosphatidylcholine (^14^C-PC). Radioactivity in the chromatographic spots corresponding to ^14^C-LPC, but not in those of ^14^C-FFA, indicates type A_1_ activity; in the opposite case, there would be type A_2_ phospholipase activity. Radioactive substrates were purchased from New England Nuclear, Boston, Mass. Each of the above (4 *μ*Ci per assay) was mixed with 1.0 mL 0.1 M sodium Tris-base buffer (pH 8.0), 2 mM CaCl_2_, 0.2% Triton X-100, and 0.27 mM phosphatidylcholine. The mixtures were sonicated with an Ultratrip Labsonic System (Lab-Line Instrument Inc., Melrose Park, IIinois), which was operated at 40 W for 60 s. The resultant emulsion was distributed in 0.5-mL aliquots and stored at –70°C until use.

The P30 fraction (10 *μ*L containing 200 *μ*g total proteins) was mixed with 10 *μ*L substrate and incubated at 36.5°C (water bath) for 2.5 h. The reaction was stopped by adding 25 *μ*L 5% trichloroacetic acid/n-butanol stop solution containing the following internal controls: rat liver free fatty acids (FFA; final concentration 1 mg/mL), egg-yolk lysophosphatidylcholine (LPC; 1 mg/mL), and egg-yolk phosphatidylcholine (PC; 0.75 mg/mL).

The hydrolysis mixtures were located drop-by-drop on the origins of 10 × 10 cm silica-gel thin-layer chromatography plates (60 mesh; Merck, Darmstadt, Germany). The plates were placed into a developing tank with a solvent system composed of chloroform:methanol:acetic acid:water (140:40:16:8 volumes). The lipid spots were developed by exposure to iodine vapor [25]. The spots belonging to PC, LPC, and FFA were identified, recovered by scratching them off, mixed with 5 mL liquid scintillation mixture (6% 2,5-diphenyloxazole/toluene) in 20-mL borosilicate vials, and quantified on a liquid scintillation counter (Packard Tri-Carb 1600TR, Beckman Instruments) [24]. All determinations were performed three times, in triplicate, and presented as mean ± SD. Specific phospholipase activity (PLAU/mg of total protein/h) was calculated by arbitrarily defining 1 unit of phospholipase (PLAU) as the hydrolysis of 1 pmol of (2-^14^C-PA)-PC.

### 2.6. Quantifications of Proteins

Protein concentrations were quantified by the Lowry method [26].

### 2.7. Virulence Test by Liver Abscess Formation

All animal work realized in this research was realized according to international ethical guidelines. All procedures were performed by a veterinarian expert in animal handling. Freshly weaned, male golden hamsters (*Mesocricetus auratus*), weighing 40-60 g [27], were anesthetized intraperitoneally with 63 mg/kg sodium pentobarbital Anestesal® (Smithkline Norden, Mexico) using a syringe with a G25 needle, so that the hamsters would remain anesthetized for one hour including a 15-min deep anesthesia. This way, suffering of the animals, was minimized. Laparotomy was realized in each hamster under aseptic conditions. The ventral lobe of the liver was inoculated with a 0.1 mL suspension of basal medium containing 10^6^ trophozoites grown in PEHPS, CLUPS, or TYI-S-33 medium (n=4). The negative control group was inoculated with parasite-free medium. After inoculation, the incision was sutured using a continuous padlock [28]. After surgery, hamsters were kept under standard laboratory conditions with water and food* ad libitum* for three days, until they were sacrificed by euthanasia with an anesthetic overdose (130 mg/kg sodium pentobarbital). After making sure that the animal had died (No breathing or heartbeat), an exploratory laparotomy was performed to evaluate liver abscess formation by direct observation and measurement of the lesion. Here to, fine cuts were made in the liberated livers of all animals with a sterile scalpel blade until liver lesions were found and could be measured.

### 2.8. Statistical Analysis

Student's* t*-test was used to compare groups with a normal distribution; a* p*-value < 0.05 was considered significant. All statistics were carried out with SPSS v18.

## 3. Results

**Figure 1:**
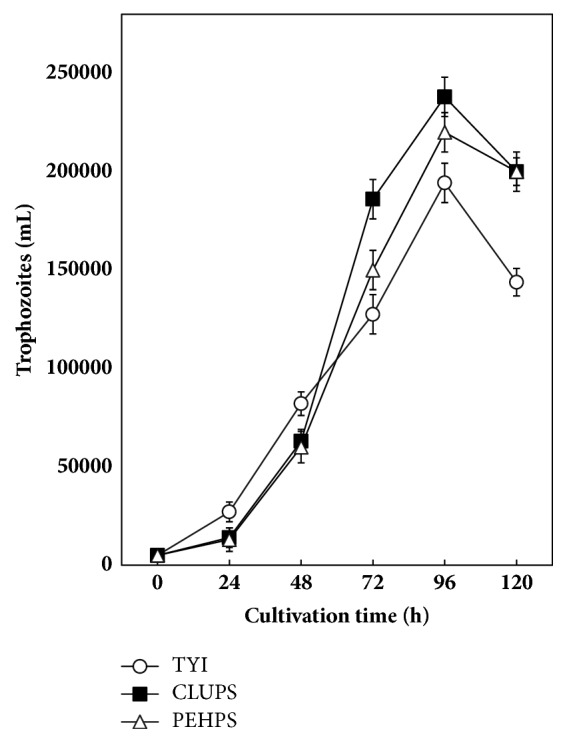
Comparative 5-day growth of* E. histolytica* trophozoites in PEHPS, TYI-S-33, and CLUPS. Results are represented as mean ± SD of quintuplicated assays.

After an adaptation lag, trophozoites of* E. histolytica* HM 1: IMSS, transferred from TYI-S-33 to CLUPS and PEHPS media, grew better in the latter 2 media at 72 and 96 h after transfer, CLUPS > PEHPS > TYI-S-33 (Figure 1). Trophozoites grown in CLUPS medium had 32% and 19% better yield than those grown in TYI-S-33 medium at 72 and 96 h, respectively, whereas the yield in CLUPS was 19% and 8% higher than the one in PEHPS at the same incubation times. CLUPS medium allowed for the shortest doubling time of growing* E. histolytica* trophozoites (21.8 h); the doubling time in PEHPS was 22.0 h and in TYI-S-33 23.2 h.

**Figure 2:**
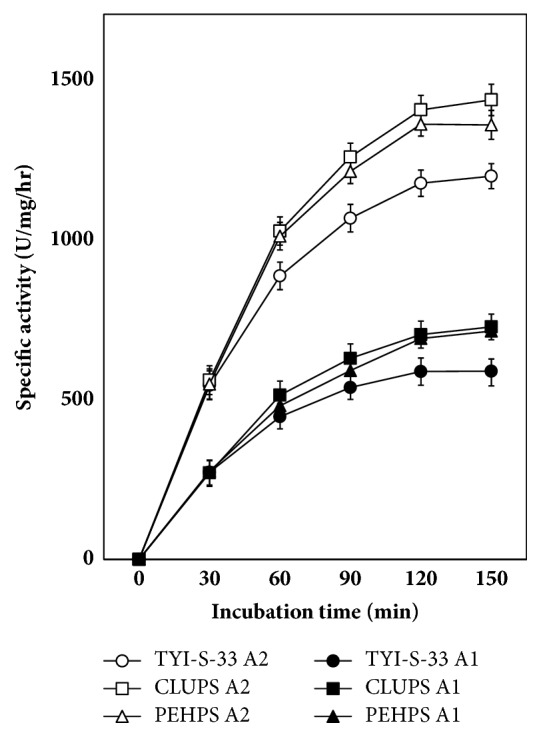
Phospholipase A1 (A1) and phospholipase A2 (A2) activity of P30 trophozoite fractions as a function of time.* E. histolytica* trophozoites were grown in PEHPS, TYI-S-33, or CLUPS; mean ± SD of quintuplicated assays.

**Figure 3:**
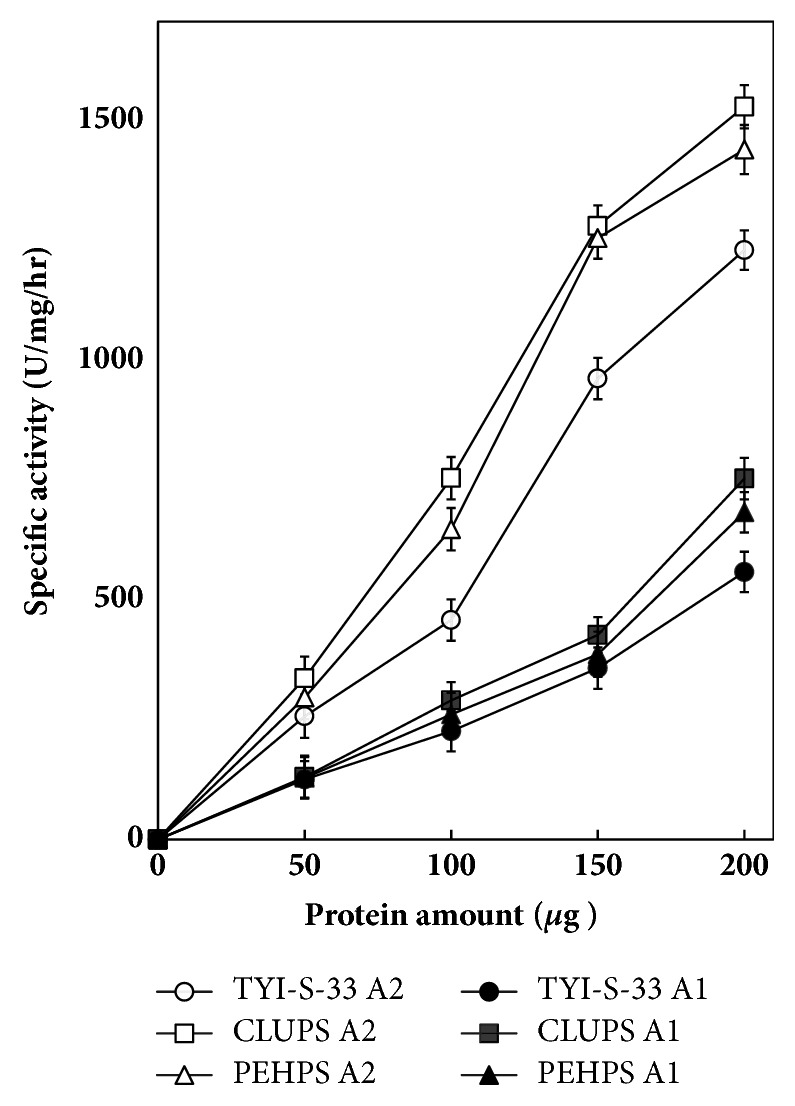
Phospholipase A1 (A1) and phospholipase A2 (A2) activity of P30 trophozoite fractions according to protein amount.* E. histolytica* trophozoites were grown in PEHPS, TYI-S-33, or CLUPS; mean ± SD of quintuplicated assays.

The radioactive products (2-^14^C-PA)-LPC and (^14^C) palmitic acid accumulated as a function of incubation time; the increase was almost linear the first 60 min, then slowed down, and reached a plateau at 90 min. Trophozoites grown in any of the three-culture media maintained the production of phospholipases A_1_ and A_2_ (Figure 2). However, the production was higher when parasites were cultured in CLUPS. At 120 min for both phospholipases A_1_ and A_2_, the difference was statistically significant between CLUPS and TYI-S-33, but not between CLUPS and PEHPS (Figure 2). Finally, the accumulation of hydrolysis products from (2-^14^C-PA)-PC increases linearly as a function of P30 protein amount in both A_1_ and A_2_ phospholipase activity (Figure 3).

All hamsters that had been inoculated with trophozoites cultured in any of the tested media showed the formation of liver abscesses. All abscesses had a diameter greater than 3 mm.

## 4. Discussion

We introduce a new culture medium (CLUPS) for the axenic growth of* E. histolytica*. CLUPS medium is a modification of the PEHPS medium [14]; the raw beef liver of PEHPS was replaced by raw beef lung in CLUPS. CLUPS was designed to avoid antibiotic remnants in beef liver. Growth curve and virulence factor analysis was expected to be similar to conventional TYI-S-33 medium or the in-house made PEHPS. Unexpectedly, we found that trophozoites grown in the CLUPS medium showed higher phospholipase activity than that the ones grown in TYI-S-33 medium [11].

At 72 h of cultivation, the trophozoite yield in CLUPS was 30% higher than in TYI-S-33. This superior performance was significant and cannot be explained by simple media variability [16]. When comparing the composition of both media, the main difference is the absence of the yeast extract in CLUPS medium. The yeast extract is considered an essential component in the culture media for amebas [29, 30]. However, we have maintained the* E. histolytica* HM-1 strain in continuous culture with yeast-extract-free PEHPS for over 25 years [14, 21]. The above proves that a yeast extract is not essential for trophozoite growth.

When we compared the growth pattern between CLUPS and PEHPS, it seems that trophozoite growth starts and ends earlier in CLUPS than in PEHPS. This retardation in PEHPS may be due to the presence of antiparasitic or antibiotic residues in the raw beef liver [18–20], which rather than growth inhibition may be the cause of a growth delay.

The production of phospholipases is the same for both media; the seemingly higher production in CLUPS was not statistically significant. So far, it seems that the CLUPS medium outperforms PEHPS medium for the culture of* E. histolytica* trophozoites; however this would have to be demonstrated by prolonged use.

The effectiveness of virulence factors in amebas is demonstrated* in vivo* by the formation of hepatic abscesses; it has even been shown that amebas increase their virulence after inoculating them in hamster liver and recovering them from lesions [31]. Although it has been mentioned that pathogenic amebas induce liver abscesses whereas nonpathogenic amebas are unable to produce them, recently it has been shown that some nonpathogenic species can produce liver abscesses [32]. We carried out an assay to demonstrate that the trophozoites of the HM1: IMSS strain grown in the CLUPS medium are capable of producing hepatic abscesses as well as those grown in PEHPS and TYI-S-33.

Alternatively, the CLUPS medium may contain factors that favor proper growth and an increased phospholipase activity. To verify if lung extracts have better factors than the other culture media, we will compare the growth and enzymatic activity of amebas cultivated in either CLUPS or PEHPS medium. At the moment no commercial medium has extracts from animal organs [10, 11].

The successful culture of this parasite in this new CLUPS medium allows for the execution of assays in an economical and stable environment. Similar to the PEHPS medium, CLUPS avoids assay variability due to interlot variability common in commercial culture media [13]. In this regard, it should be mentioned that a large batch of concentrated organ extract suffices for over a year's supply for in-house prepared medium [14].

The reliable cultivation of* E. histolytica* in CLUPS medium allows us to investigate the biology of this parasite, to study its virulence factors, to develop better diagnostic methods, and to test new drugs for better treatments.

In conclusion, we consider that the CLUPS medium is a superior alternative culture medium, which is both economic and free of the interlot variability present in commercial TYI-S-33.

## Figures and Tables

**Table 1:** Composition of the culture media, PEHPS, TYI-S-33, and CLUPS.

**Component**	**Brand**	**PEHPS [9]**	**TYI-S-33**	**CLUPS**
Liver and pancreas extract (mL)	In-house	250	None	None
Lung and pancreas extract (mL)	In-house	None	None	250
Liver infusion broth (g)	BD Difco	None	10	None
Casein peptone (g)	BD Bioxon	10	20	10
Ascorbic acid (g)	Sigma Aldrich	0.2	1	1
Cysteine (g)	Sigma Aldrich	1.0	1	1.26
Glucose (g)	Tecnica quimica	6	10	6
K_2_HPO_4_ (g)	Baker	1	1	1
KH_2_PO_4_ (g)	Monterrey	0.6	0.6	0.6
Sodium chloride (g)	Baker	None	3.3	None
Ferric citrate (g)	Sigma Aldrich	None	0.02	None
Deionized water		Up to 1L	Up to 1L	Up to 1L
